# Understanding natal emigration in group‐living carnivores

**DOI:** 10.1111/1365-2656.70330

**Published:** 2026-07-30

**Authors:** Kathleen M. Petersen, Joel M. Tebbenkamp, Matthew A. Mumma, David E. Ausband

**Affiliations:** ^1^ Idaho Cooperative Fish and Wildlife Research Unit University of Idaho Moscow Idaho USA; ^2^ Idaho Department of Fish and Game Wildlife Health and Forensics Laboratory Eagle Idaho USA; ^3^ Idaho Department of Fish and Game State wildlife management agency Boise Idaho USA; ^4^ U.S. Geological Survey, Idaho Cooperative Fish and Wildlife Research Unit University of Idaho Moscow Idaho USA

**Keywords:** *Canis lupus*, cooperative breeding, emigration, genetic sampling, grey wolf, harvest management, natal dispersal

## Abstract

1. Dispersal is a critical ecological and evolutionary process, yet the drivers of individual variation in emigration, particularly under human‐modified conditions, remain relatively understudied. We investigate the determinants and patterns of emigration in a managed population of cooperative breeders using genetic samples from harvested individuals.
2. We used a novel genetic approach to identify natal emigration through sibship analyses of DNA from harvested grey wolves (*Canis lupus*), a cooperative‐breeding species, and evaluated how prey biomass, wolf density, human‐caused mortality and sex influenced natal emigration across Idaho, USA, from 2017 to 2023.
3. From our analysis of 182 sibling groups (representing packs), we evaluated natal emigration status for 224 individuals. Of these, 86 were classified as emigrants and 138 as non‐dispersers. We assessed how covariates influenced the probability of emigration and evaluated sex differences in apparent dispersal distance.
4. We found no sex differences in emigration propensity or apparent dispersal distance. Emigrants dispersed an average of 81.8 km (range: 30.0–338.9 km) from their inferred natal territories. Emigration was more likely from territories with higher prey biomass and lower wolf density, while human‐caused mortality had no detectable effect.
5. We found that ecological drivers, such as prey availability and population density, have a stronger effect on the propensity to emigrate than harvest intensity. These results highlight the importance of resource availability and population dynamics in shaping dispersal patterns in a cooperative breeder. Our study used a novel genetic method to assess natal emigration in a group‐living carnivore and developed a framework broadly applicable to social systems where kin structures can be traced to dispersers. Our findings identify landscape‐scale conditions that influence dispersal and can inform the management of wild populations.

## Graphical abstract

Dispersal is a key ecological and evolutionary process, yet drivers of variation in emigration under human‐modified conditions remain understudied. Using a novel genetic approach applied to harvested grey wolves in Idaho, USA, we found that prey availability and population density shape natal emigration patterns in a cooperative breeder.

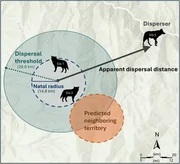

## 1 INTRODUCTION

Dispersal is a key demographic process that enables populations to maintain genetic diversity and persist over time (Clobert et al., 2012; Wright, 1951). Natal emigration is the first and often most consequential stage of dispersal, as it involves the decision to leave a natal territory. Individual traits, as well as social and environmental contexts, shape natal emigration. At the individual level, emigration can vary with age, body condition and other intrinsic traits (Clobert et al., 2012; Cote & Clobert, 2007). Emigration can also be constrained by trade‐offs between dispersal capacity and reproductive prospects (Clobert et al., 2012; Maag et al., 2018). Social conditions, such as the risk of inbreeding (Nelson‐Flower et al., 2012), competition for mates or resources (Koenig & Pitelka, 1981), and variation in population density or sex ratio can further shape emigration decisions (Ausband, 2022b; Bonte et al., 2012; Doolan & Macdonald, 1996; Greenwood, 1980; Komdeur et al., 2017; Maag et al., 2018). Environmental factors, including local habitat quality and access to key resources, such as food or space, also influence whether individuals leave their natal territory (Bates et al., 2006; Bonte et al., 2008; Bowler & Benton, 2009).

In species that live in social groups and breed cooperatively, some individuals may delay dispersal (Koenig et al., 1992). Groups of cooperative breeders typically consist of a dominant breeding pair and subordinate helpers. Helpers either queue for future breeding opportunities or forego breeding altogether to assist in rearing young (Clutton‐Brock et al., 2016). In these groups, individuals can remain in their natal territory to help or disperse to seek breeding opportunities elsewhere (Clutton‐Brock et al., 2016). Philopatry, or the decision to stay within the natal territory, is integral to the complex social structure of cooperative breeders. Remaining in the natal group provides important benefits to fitness. Philopatry can increase inclusive fitness by allowing individuals in a group to gain indirect fitness benefits through kin selection (Hamilton, 1964; Wild & Taylor, 2014). Staying can also improve access to shared resources and food availability through group living (Groenewoud & Clutton‐Brock, 2021) and support territory defence that benefits the entire group (Clutton‐Brock, 2006). Additionally, group membership can increase reproductive success for individuals who remain in the natal group, but it may delay access to breeding opportunities (Sparkman et al., 2011; Wild & Taylor, 2014). However, these benefits are not without costs. Staying in the group can lead to heightened kin competition among group members (Cote & Clobert, 2010; West et al., 2002), particularly for resources and future breeding positions, which may eventually outweigh the benefits of philopatry (Le Galliard et al., 2005).

Given these trade‐offs, the decision to disperse represents an alternative fitness strategy to secure reproductive opportunities elsewhere. Dispersal offers potential benefits, including reduced kin competition within the natal group and access to new breeding opportunities elsewhere (Clobert et al., 2009; Lambin et al., 2001). However, dispersal also carries substantial costs. Individuals face increased risks of mortality, including encounters with other wolves and starvation, while navigating unfamiliar landscapes and travelling independently to establish or join a new group (Bonte et al., 2012; Pusey & Packer, 1987). Although dispersal may provide earlier breeding opportunities, it can come at the cost of increased mortality (Bonte et al., 2012; Boyd & Pletscher, 1999; Clutton‐Brock, 2006). These complexities reflect a fundamental trade‐off between alternative reproductive strategies to stay or leave a natal territory. As a result, dispersal behaviour is especially difficult to predict in cooperative breeders, where these dynamics complicate individual decision‐making.

Adding to this challenge, dispersal characteristics vary widely within and across populations of the same species (Bowler & Benton, 2009; Clobert et al., 2009), making it difficult to draw broad conclusions about dispersal strategies in group‐living animals. This extensive variation, combined with inconsistent methodologies and limited data from wild populations, has hindered identification of the proximate causes of natal emigration (Matthysen, 2012; Morales‐González et al., 2021). Recent advances in non‐invasive genetic sampling now make it possible to reconstruct genealogical relationships with greater accuracy. By identifying family groups, genetic data provide a powerful tool for answering questions about emigration. Here, we use a novel genetic approach to assess natal emigration in a cooperatively breeding species, providing a framework broadly applicable to social systems where kin structures can be traced to dispersers. Our approach could be informative in species with structured social groups, such as matriarchal or patriarchal primate societies (Silk, 2005) and family groups of cooperative‐breeding mammals and birds (Stacey & Koenig, 1990).

Grey wolves (*Canis lupus*) in Idaho, USA, present a unique system for studying emigration in cooperatively breeding carnivores due to their complex social structures, long‐distance movements and demographic pressures in human‐influenced landscapes. Wolves typically live in multigenerational family groups with reproduction dominated by a breeding pair (i.e. parents) although subordinates can occasionally reproduce (Ausband, 2018; Mech & Boitani, 2003). Individuals generally disperse from their natal groups at ages 1–4 years to seek breeding opportunities elsewhere, and such behaviour is generally male‐biased in many wolf populations (Ausband, 2022b; Jimenez et al., 2017; Kojola et al., 2006). Both male and female wolves can disperse over 850 km (Boyd & Pletscher, 1999; Ciucci et al., 2009; Mech & Boitani, 2003), with dispersal in North America peaking in late fall and early winter during the mating season (Mech & Boitani, 2003). Understanding dispersal in wolves is challenging due to their large home ranges and long‐distance movements (Jimenez et al., 2017). However, understanding these movements is critical because limited dispersal can constrain population growth (Morales‐González et al., 2021; Packard & Mech, 1980), while immigration from neighbouring populations helps offset high mortality and maintain genetic diversity, even under intensive harvest pressure (Adams et al., 2008; Ballard et al., 1987; Fuller et al., 2003).

Although dispersal has been studied well in cooperative breeders, the effects of harvest on dispersal are not well understood, and factors, such as social status, age and resource availability, do not fully explain wolf emigration patterns (Morales‐González et al., 2021). The Idaho Department of Fish and Game (IDFG) uses annual harvest to regulate wolf populations. Variation in harvest intensity among groups provides a natural experiment to examine the long‐term effects of human‐caused mortality on natal emigration.

Our objective was to understand how ecological, demographic and human factors contribute to the dispersal of wolves from natal territories. We identified dispersers using genetic samples from grey wolves harvested in Idaho and tested the influence of prey biomass, population density, sex and harvest on the probability of natal emigration (Table 1). First, we hypothesized that natal emigration would be sex‐biased (McNUTT, 1996; Trochet et al., 2016). Consistent with other studies, we predicted that male wolves would emigrate more frequently and disperse farther than females because males more often explore new territories, whereas females tend to remain in natal groups to queue for breeding opportunities (Ausband, 2022a; Fenner et al., 2025). Second, we hypothesized that natal emigration would exhibit density dependence, where emigration probability changes in response to population density in the natal territories and surrounding area (Blanco & Cortés, 2007; Travis et al., 1999). Specifically, we predicted a non‐linear relationship, with emigration lowest at intermediate densities where cooperation and breeding opportunities within natal territories are maximized (Hoogland, 2013; Kokko & Lundberg, 2001; Maag et al., 2018). We also predicted increased emigration at low densities due to reduced benefits of cooperation and at high densities due to intragroup and kin competition limiting inclusive fitness (Hamilton & May, 1977; Hoogland, 2013). Third, consistent with the ecological constraints hypothesis, we hypothesized that declining resource availability would increase emigration probability. Ecological constraints theory proposes that individuals are more likely to disperse when ecological constraints make staying in a natal territory less beneficial (Emlen, 1982; Stacey & Ligon, 1987). We used prey biomass as an index of resource availability in the broader area surrounding natal territories. We predicted that wolves in natal territories with lower prey biomass would emigrate more frequently than those in resource‐rich areas (Fuller et al., 2003; Gese et al., 1996; Messier, 1985). Finally, we hypothesized that the external factor of human‐caused mortality would alter regional social and demographic conditions relevant to natal dispersal. We predicted that areas with higher harvest intensity at the Game Management Unit scale would increase emigration by reducing population density and creating breeder vacancies, thereby altering social structure and increasing breeding opportunities among packs (Ausband, 2022b; Cassidy et al., 2023; Lazaro‐Perea et al., 2000).

**TABLE 1:** Predictions for grey wolf (*Canis lupus*) natal emigration as a function of relative wolf density, prey abundance, sex and wolf harvest. The scale at which variables were estimated is indicated.

Variable	Emigration	Scale considered	Rationale/source
Male	+	Individual	Higher dispersal and more likely to breed outside natal group (Ausband, 2022b; Jimenez et al., 2017)
Female	−	Individual	More likely to inherit breeding role in natal group (Ausband, 2022b)
Medium wolf density	−	686 km^2^ scaled to wolves/1000 km^2^	Cooperative benefits reduce dispersal; fewer vacancies (Clutton‐Brock, 2002; Kokko & Lundberg, 2001); Lowest dispersal in meerkats at medium density (Maag et al., 2018)
Low wolf density	+	686 km^2^ scaled to wolves/1000 km^2^	Fewer cooperative benefits; more territory vacancies promote dispersal (Hoogland, 2013; Maag et al., 2018)
High wolf density	+	686 km^2^ scaled to wolves/1000 km^2^	Increased interpack conflict and kin competition drive dispersal (Cote & Clobert, 2007; Maag et al., 2018)
Low prey	+	GMU scaled to biomass/100 Morales‐González	Resource scarcity promotes dispersal to improve access to prey and territory (Fuller et al., 2003, Gese et al., 1996, Mech et al., 1998, Messier, 1985, Peterson et al., 1998)
High harvest	+	GMU scaled to wolves/1000 km^2^	Female removal increases helper dispersal due to reduced kin facilitation (Hoogland, 2013), Harvest creates breeding vacancies (Ausband, 2022a)

## 2 METHODS

### 2.1 Study area

Idaho is located west of the continental divide in the United States, and the study area encompassed the entire state. Idaho covers 216,632 km^2^ and includes substantial variation in elevation, climate and habitat. Elevation ranges from 217 to 3859 m. Climate varies widely across the state, with temperatures ranging from approximately −7 to 29°C across seasons and regions. Annual precipitation ranges from approximately 64 to 127 cm in northern Idaho and 30.5 to 63.5 cm in central and eastern Idaho (Western Regional Climate Center, 2022). Northern Idaho is characterized as an inland temperate rainforest, whereas central and eastern Idaho include transitional vegetation types between coniferous forests, sagebrush steppes and high desert areas bordering agricultural land. Southern Idaho is dominated by sagebrush steppes and arid canyons, interspersed with fragmented agricultural and urban landscapes. These broad climatic and habitat gradients correspond to differences in the distribution of ungulate prey in the state. Across Idaho, wolves prey on large and medium ungulates, including moose (*Alces alces*), white‐tailed deer (*Odocoileus virginianus*), mule deer (*O. hemionus*) and elk (*Cervus canadensis*), with the presence and abundance of these prey species varying across the state.

### 2.2 Harvest methods

The Idaho Department of Fish and Game uses regulated wolf harvest through hunting and trapping as a management tool across Idaho. Wolves of any age can be legally harvested and harvest intensity varies across the state. During the study, harvest regulations varied in time and space. During the initial years, the length of hunting and trapping seasons differed between private and public land and across game management units. Furthermore, some Game Management Units allowed unlimited take; other GMUs closed the season once quotas were reached. Through time, wolf harvest was increasingly liberalized on both private and public land via extended seasons, elimination of quotas and additional methods of take. IDFG and the Wolf Depredation Control Board ‘provide financial support to a private third‐party entity that has reimbursed wolf hunters and trappers for their documented harvest‐related expenses’ (Idaho Department of Fish and Game, 2023). Additional wolves were removed to minimize wolf‐livestock conflicts. Across 2017–2023, mean wolf harvest, including wolf‐livestock removals, was 452 wolves, with a low of 379 in calendar year 2017 and a high of 515 in calendar year 2021.

### 2.3 Sampling

All harvested wolves were required to be checked‐in with Idaho Department of Fish and Game, and biological samples were collected from the majority of harvested wolves. Although samples were also collected from other sources of human‐caused mortality (e.g. roadkill and wolf‐livestock conflict), we used samples from harvested wolves (i.e. hunting and trapping) because harvest is the predominant source of wolf mortality in Idaho and can be directly influenced by harvest regulations set by managers. During mandatory harvest check‐in, IDFG personnel removed a premolar tooth for cementum ageing (Matson's Laboratory, Manhattan, MT, USA) and collected tissue or rinsed the tooth for genotyping. We used harvest samples to identify sibling groups, classify emigration status and estimate wolf harvest. No institutional animal care and use approval was required because this study relied exclusively on existing biological samples and associated data collected by the Idaho Department of Fish and Game during mandatory harvest check‐in and routine management activities.

### 2.4 Laboratory methods

We used Genotyping‐in‐Thousands (GT‐seq; Campbell et al., 2015) to amplify 341 single‐nucleotide polymorphism (SNP) and microhaplotype loci developed for grey wolves in Idaho (Delomas et al., 2023). Within this panel, 320 loci were optimized for individual identification and relationship inference based on high heterozygosity, while 10 distinguished western coyotes (*C. latrans*) and wolves, nine differentiated wolves and domestic dogs (*C. familiaris*) and one locus determined genetic sex. Sequencing was performed on an Illumina NextSeq 500 system using 150‐bp paired‐end reads, with technical replicates to ensure accuracy and control for errors. For additional details on panel development and methodology, refer to Delomas et al. (2023).

### 2.5 Emigrant identification

We inferred sibling relationships among harvested wolves from 2017 to 2023 using program COLONY (Jones & Wang, 2010). We matched inferred siblings back to birth years 2017 to 2022 and included wolves harvested in 2023 that were born in earlier years. For each birth year, we estimated allele frequencies using all individuals born before the focal year, regardless of harvest status. For sibship analyses, we included 340 loci (e.g. all but sex marker). We specified COLONY settings for a genotyping error rate and false allele rate of 0.005, a dioecious mating system with male and female monogamy and allowed for inbreeding. The analyses excluded clone inference and removed sibship priors, which allowed COLONY to infer sibling groups solely from genetic data without assumptions about family structure or size. We included sibship scaling to reduce false positive sibship assignments (Wang, 2013) and to help avoid assigning unrelated individuals as siblings when sibling groups are large. Although this is less critical for small litter sizes, we used this setting to improve consistency between runs and provide a conservative approach that prevented dispersal assignments between individuals not from the same litter. To assess assignment consistency, we ran COLONY 10 times for each birth year using different random number seeds and retained sibling relationships only when they were identified in at least 8 out of 10 runs. We only retained sibling relationships with a probability of inclusion >0.90, which also reduced the likelihood of false positive sibship assignments.

We compared harvested wolves between 2017 and 2023 and assigned them into the inferred sibling groups. We used age‐at‐harvest (cementum age) to assign year of birth and used the sibling relationships to delineate sibling groups within each birth year. We restricted analyses to individuals assigned to the same birth year to further reduce the likelihood of incorrectly assigning closely related individuals from different generations (e.g. parent–offspring pairs) as siblings.

We assigned harvest latitude and longitude using reported kill locations provided by hunters and supplemented these with biologist‐derived spatial inference within an average wolf territory cell (686 km^2^) when exact locations fell within larger areas; see Methods S1 for additional details. To identify potential dispersers that emigrated outside of their natal territory, we focused on wolves 1‐year‐of‐age or older and compared their harvest locations with those of younger siblings aged 0–12 months. We assumed that the wolves harvested at 0–12 months had not yet dispersed; this is consistent with previous work showing that 96% of the wolves in this system disperse at 1 year or older (Jimenez et al., 2017); thus, when a wolf >1‐year‐of‐age was harvested far from its siblings that were harvested at <1 year‐of‐age, we identified it as a natal emigrant.

We classified an emigration event using a distance threshold of 29.6 km (Figure 1, Methods S2), which is equivalent to twice the radius of the mean size of wolf territories in Idaho (686 km^2^; Ausband, 2016). Although we cannot assess the exact stage of dispersal at harvest to determine whether the wolves were still transient or settled in a new territory, our conservative distance threshold (29.6 km) suggests that the individuals were no longer within their natal territory. We refer to the distance between the natal territory and the harvest location as the apparent dispersal distance but acknowledge that the true dispersal stage at the endpoint cannot be confirmed. Given uncertainty in the true dispersal endpoint, we assumed that harvest locations likely approximate actual settlement points because the transience stage of wolf dispersal is relatively brief, with a mean duration of 5.5 months in the Rocky Mountains (Jimenez et al., 2017).

**FIGURE 1:**
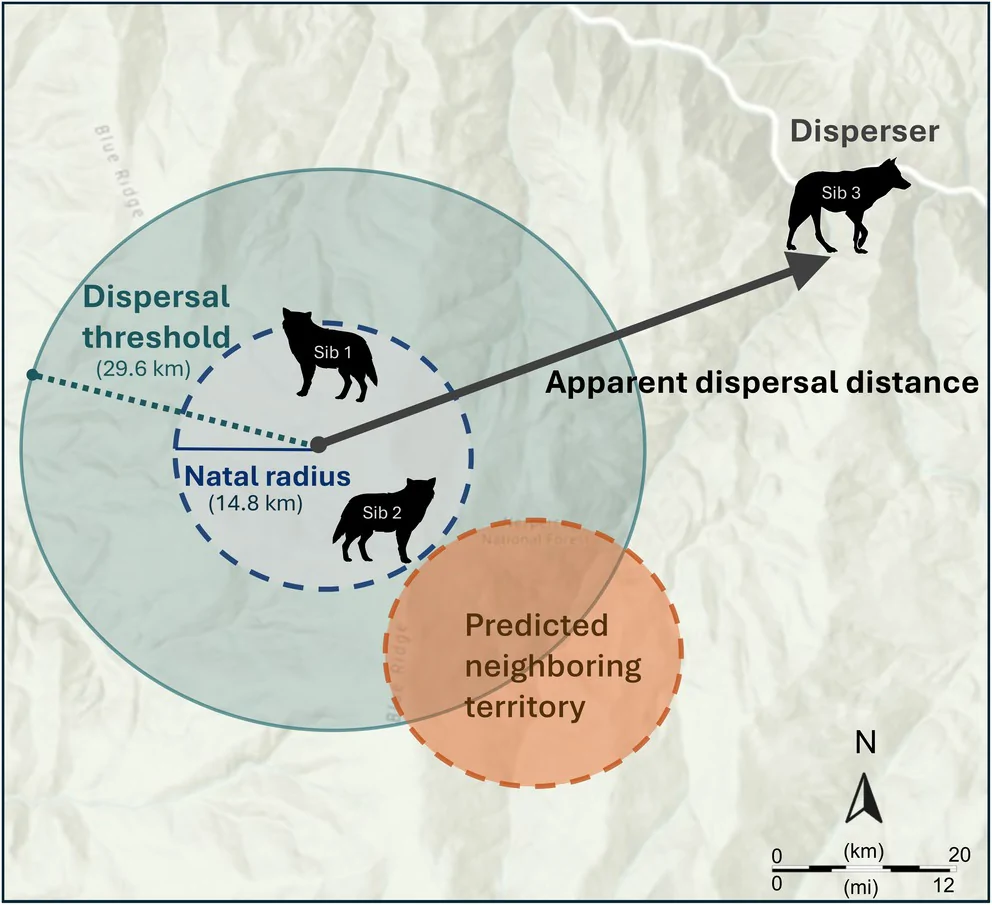
Schematic of the study design used to identify dispersers. The central circle represents a natal territory with a radius of 14.8 km, based on the average wolf territory size in Idaho (686 km^2^; Ausband et al., 2014). The large outer circle represents the dispersal threshold at twice this radius (29.6 km). Individuals detected beyond this distance were classified as emigrants from the natal territory. The grey line indicates the apparent dispersal distance measured from the natal territory centroid to the harvest location of the disperser. Wolf icons labelled Sib1–3 represent siblings born in the same year from the same group. The orange circle illustrates a predicted neighbouring territory.

We measured the apparent dispersal distances using two approaches. When only one sibling <1‐year‐of‐age was harvested, we measured the distance between the <1‐year‐old sibling and any siblings harvested in future years (>1‐year). When multiple siblings <1‐year‐of‐age were harvested, we determined the centroid of harvest locations for <1‐year‐old siblings and measured the distance between the centroid and any siblings harvested in future years (>1‐year) (Figure 2). The harvest locations of the >1‐year‐old siblings were treated as the assumed dispersal locations.

**FIGURE 2:**
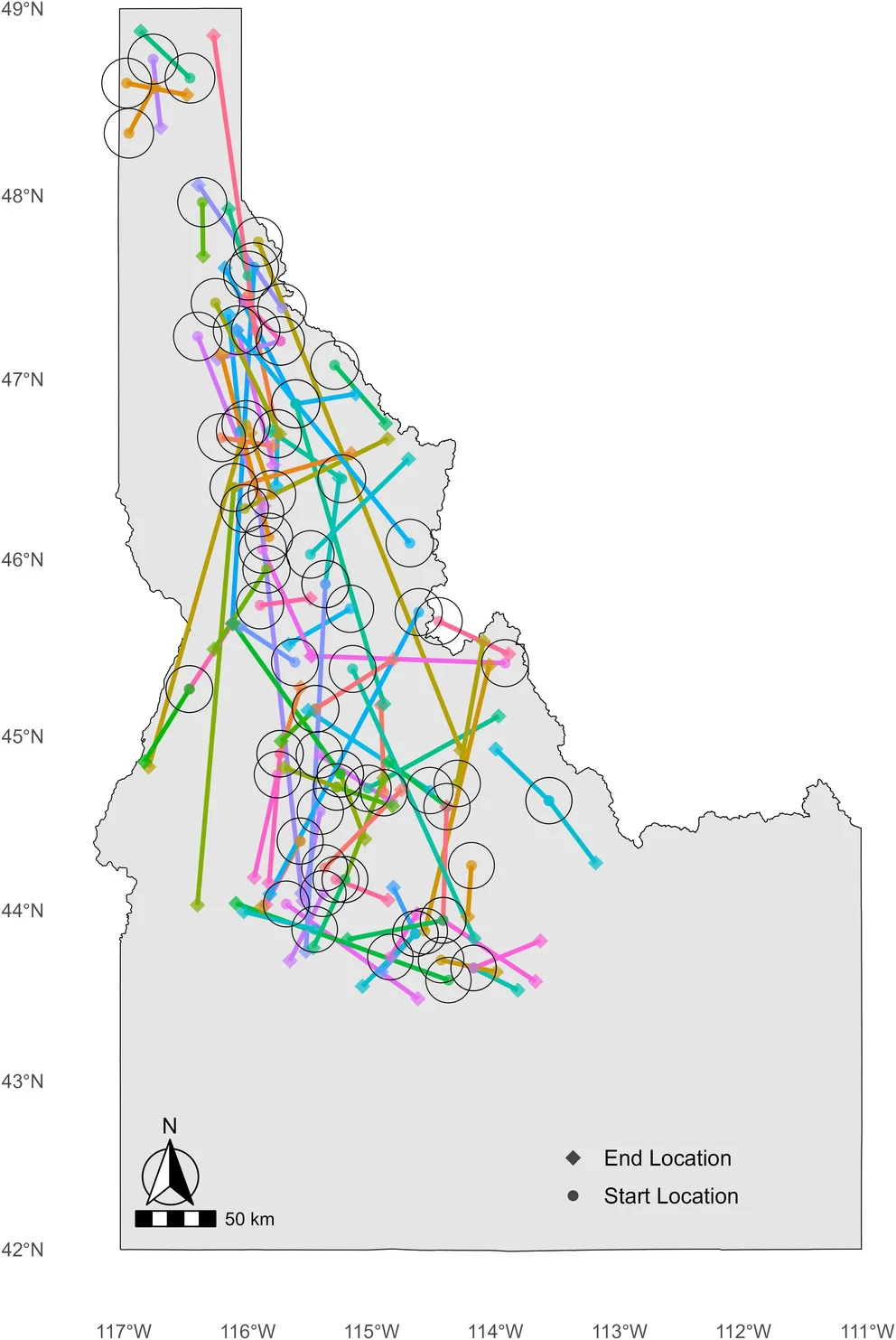
Natal emigration events documented for grey wolves (*Canis lupus*) harvested in Idaho (2017–2023). The map shows start locations (circle symbols) representing the centroids of sibling wolves <1 year old. Black circles surrounding these points represent the radius of predicted natal territories. Natal emigration events of wolves ≥1 year old are displayed as lines extending from natal groups to assumed dispersal locations, with end (harvest) locations shown as diamonds. Each line is colour coded to distinguish individual emigrants.

### 2.6 Covariates

#### 2.6.1 Wolf relative density

To assess the influence of wolf density on emigration probability, we first estimated relative wolf abundance at the predicted natal territory. We used IDFG camera trap data collected from 2017 to 2022 (Figure S6). Trail cams were deployed annually within viable wolf habitat throughout Idaho by IDFG (Ausband et al., 2023). The sampling design placed one camera per 686 km^2^ grid cell (the average wolf territory size in Idaho; Ausband et al., 2014), typically along roads or trails, during July and August of each year. Using images from the cameras, we fit N‐mixture models to estimate relative wolf abundance while accounting for imperfect detection (Royle, 2004). The abundance model included forest cover (percentage of tree cover within each grid cell) and the proportion of each grid cell located in Idaho as covariates. The detection model included operational days (Methods S3). We based model selection on the Akaike Information Criterion (AIC; Akaike, 1974) and assessed model fit using a goodness‐of‐fit test with 1000 bootstrap iterations. We conducted all analyses using the R statistical software (R Core Team, 2025) using camtrapR (Niedballa et al., 2016) and unmarked R packages (Fiske & Chandler, 2011). We adjusted the relative abundance estimate derived from the N‐mixture model to a relative density by dividing it by the area of the camera grid cells. Then, we converted it from the grid cell scale (that is, 686 km^2^) to a per‐1000 km^2^ scale to facilitate comparison with existing literature.

The best‐supported N‐mixture model for estimating wolf abundance in all years used a negative binomial distribution, which provided the best fit among candidate models based on Akaike's information criterion (AIC; Akaike, 1974).

#### 2.6.2 Prey biomass

We evaluated the effect of prey biomass on wolf dispersal from natal territories by quantifying biomass using IDFG ungulate harvest data. Data included elk (*Cervus canadensis*), mule deer (*Odocoileus hemionus*) and white‐tailed deer (*O. virginianus*) from Game Management Units (GMU) that allowed harvest of the general season. To account for differences in prey availability, we calculated the catch per unit effort for each species by dividing the number of animals harvested by hunting effort days. We combined these values and adjusted them using species‐specific biomass weights (Greer & Howe, 1964; Mackie, 1964; Silver et al., 1959) to estimate total prey biomass in kg/100 km^2^/hunting day (Methods S4, Figure S7).

#### 2.6.3 Wolf harvest

To assess how wolf harvest may drive dispersal from natal territories, we calculated harvest intensity by dividing the number of wolves harvested per Game Management Unit and year by the area of the respective GMU, then scaling the result to 1000 km^2^ (Methods S5, Figure S8). These estimates were based on the complete statewide harvest of all wolves, not just the sibling subset data used to measure dispersal. This metric provided a comparable measure of harvest intensity across GMUs in Idaho.

### 2.7 Statistical analysis

To examine the potential factors that influence wolf emigration from natal territories, we built generalized linear models (GLM) with a binomial distribution and a logit link function using program R (R Core Team, 2025). The response variable was the emigration status (binary: 1 = emigrated, 0 = non‐emigrated). We used predictor variables from natal territories that included sex, wolf density/1000 km^2^ at the corresponding grid cell, prey biomass/100 km^2^ and wolf harvest/1000 km^2^ at the corresponding Game Management Unit, and assigned all variables for the birth year of all potential dispersers aged ≥1 year. We also *z*‐standardized all continuous predictors. The pairwise correlations between the predictors were weak (*r* < 0.3) and the variance inflation factors of the models were ~1, indicating that there was no significant multicollinearity.

We attempted to fit generalized linear mixed‐effects models (GLMM) using the lme4 package (Bates et al., 2015) in R (R Core Team, 2025). The models included random effects for sibling group to account for dependency among siblings from the same litter, and for year to account for dependency among individuals born in the same year. However, both models overfit due to insufficient random‐effects levels and number of observations. Therefore, we proceeded with the GLM. We evaluated candidate models using AIC, summarized in Table 2. Model fit was evaluated using standardized residual diagnostics implemented in the DHARMa R package (Hartig, 2022). In addition, we fit a linear model to examine differences in emigration distances based on sex.

**TABLE 2:** Candidate models for predicting natal emigration of grey wolves (*Canis lupus*) in Idaho, USA, from 2017 to 2022. Models include ecological and demographic predictors, such as prey biomass, wolf relative density, wolf harvest and sex.

Model	*K*	*LL*	AIC	ΔAIC_c_	AIC*w*_*i*_
Prey biomass + wolf density + sex^a^	4	−136.80	281.79	0.00	0.39
Prey biomass + wolf density	3	−137.86	281.84	0.05	0.38
Prey biomass + wolf density + wolf harvest + sex	5	−136.64	283.57	1.78	0.16
Prey biomass + wolf density + wolf density^2^ + Wolf harvest + sex	6	−136.52	285.43	3.64	0.06
Null	1	−145.32	292.66	10.87	0.00

^a^ Indicates most supported model, AUC*w*_*i*_ = 0.39.

## 3 RESULTS

Across the study area, the relative wolf density averaged 10.45 wolves/1000 km^2^ (range = 0.27–95.81). The mean biomass of the prey at the GMU level was 0.47 kg/100 km^2^ (range = 0.31–3.73). The mean wolf harvest at the GMU level was 5.33 wolves/1000 km^2^ (range = 0.49–16.94). Between 2017 and 2023, we sampled 182 sibling groups that comprised 595 individual wolves. Of these, 224 individuals were >1‐year‐old and assessed to determine whether they emigrated beyond their natal territory. We identified 86 natal emigrants and 138 non‐dispersers. Natal emigrants exhibited a mean apparent dispersal distance of 81.8 km but varied widely in their apparent dispersal distances (SD = 23.7 km, range = 30.0–338.9 km). Among dispersers, mean apparent dispersal distance was comparable between sexes, with males dispersing 81.9 ± 69.8 km (*n* = 38) and females dispersing 81.8 ± 58.2 km (*n* = 48). We did not find support that apparent dispersal distance varied by sex (male vs. female *β* = 0.08, SE = 0.07, *p* = 0.25; Table S12), indicating that males and females dispersed similar distances.

The best‐supported GLM predicting wolf emigration included prey biomass, wolf density and sex. Two additional models (one without sex covariate and another with harvest covariate included) received equal support (ΔAIC_c_ ≤2) but all three top models included prey biomass and wolf density. The top model (AIC*w*_*i*_ = 0.39) included sex (Table 2); however, another candidate model excluding sex received similar support (ΔAIC_c_ = 0.05), indicating that the inclusion of sex does not substantially improve model support. For the top model, scaled residuals were uniformly distributed (Kolmogorov–Smirnov test, *p* = 0.94), showed no evidence of outliers (outlier test, *p* = 1) and did not exhibit over‐ or under‐dispersion (dispersion = 1.01, *p* = 0.90). Quantile tests and residual plots did not show systematic deviations from model assumptions between fitted values or covariates (*p* = 0.92). Together, these diagnostics indicate a good overall fit of the model. The model had an area under the curve score of 0.65, indicating modest predictive ability (Hanley & McNeil, 1982). Although we hypothesized that wolf dispersal might exhibit a non‐linear relationship with density, including a quadratic term for wolf density did not improve model fit (ΔAIC_c_ = 3.64; Table 2), so the simpler linear model was considered most supported.

The results of the best‐supported model predicting wolf emigration (Table 3, Table S11) indicated that prey biomass positively influenced emigration probability (*β* = 0.35, SE = 0.15, *p* = 0.020). A one standard deviation increase in standardized prey biomass corresponded to 1.42 times greater odds of emigration (95% CI = 1.05–1.84; Figure 3). In contrast, wolf density negatively affected emigration (*β* = −0.56, SE = 0.20, *p* = 0.006), such that a one standard deviation increase in wolf density corresponded to 43% lower odds of emigration (95% CI = 0.38–0.85; Figure 3). Sex did not significantly influence the probability of emigration (male vs. female: *β* = 0.42, SE = 0.29, *p* = 0.147, 95% CI = 0.87–2.72). Wolf harvest was included in a model within ΔAIC <2 (*β* = −0.087, SE = 0.16, *p* = 0.578, 95% CI = 0.72–1.18), but this model was not top‐supported.

**TABLE 3:** Covariates from the most supported generalized linear model predicting grey wolf (*Canis lupus*) emigration in relation to prey biomass, wolf relative density, wolf harvest and sex in Idaho, USA, from 2017 to 2022. The table provides estimated coefficients (*β*), standard errors (SE) and *p*‐values (*p*) for each covariate.

Covariate	*β*	SE	*p*
Intercept	−0.75	0.22	<0.001
Prey biomass	0.35	0.15	0.020
Wolf density	−0.56	0.20	0.006
Sex	0.42	0.29	0.147

**FIGURE 3:**
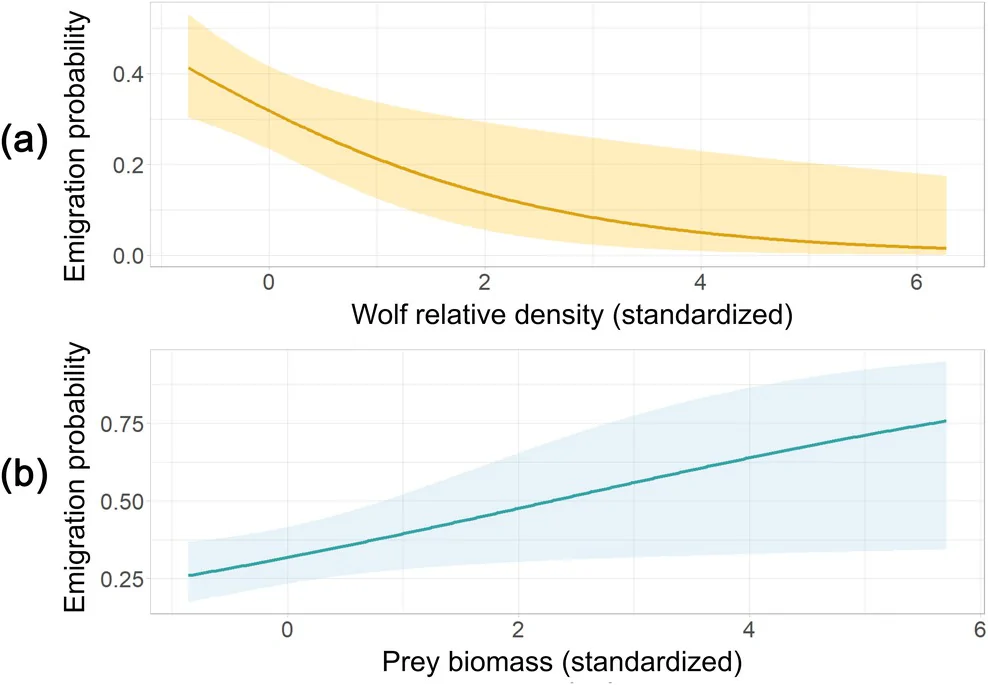
Effects plots for significant predictors of grey wolf (*Canis lupus*) emigration probability in Idaho, USA, from 2017 to 2022. Panel (a) shows the estimated effect of relative wolf density, and Panel (b) displays the estimated effect of prey biomass. Variables are *z*‐standardized and plotted with all other variables held at their mean values. Confidence intervals are displayed at the 95% level.

## 4 DISCUSSION

We investigated predictors of individual variation in dispersal in a managed population of cooperative breeders using genetic harvest samples and identified key factors associated with probability of emigration. Our findings suggest that ecological and social dynamics, such as prey availability and population density, have a stronger effect on emigration propensity than harvest intensity (at the spatial scales used in our analyses). These results highlight the interplay between resource availability, population dynamics and competition in shaping dispersal patterns in grey wolves.

As the density of wolves increased, the probability of emigration decreased, indicating that dispersal was negatively associated with density in our study (Table 3). This pattern may reflect the benefits of remaining with a familiar social group, such as siblings, in high‐density areas where competition and risks associated with dispersal are increased (Jimenez et al., 2017; Sells et al., 2022). Wolves, as cooperative breeders, are influenced by kin selection, which can favour staying and helping rather than dispersing solely to maximize individual fitness (Clutton‐Brock, 2006; Sparkman et al., 2011). On the contrary, in low‐density natal territories, limited opportunities for cooperative breeding can drive higher emigration probabilities (Hoogland, 2013). Wolves are fiercely territorial, often defending their territories through aggressive encounters with outsiders, which can lead to fatal conflicts, making dispersal a high‐risk endeavour (Benson & Patterson, 2013; Cubaynes et al., 2014). Dispersal plasticity likely moderates these patterns, allowing wolves to adapt their dispersal decisions to environmental and social conditions (Clutton‐Brock, 2006; Koenig et al., 1992). The concept of ‘social fence’ theory (Lambin et al., 2001) suggests that when the population density of neighbouring groups is low, emigration from a central group is possible because there are opportunities for individuals to establish territories or join other groups. However, as neighbouring densities increase, these opportunities diminish because neighbouring groups become socially ‘impermeable’, effectively creating a barrier to emigration (Stamps, 2001). Under these circumstances, it is still beneficial to stay and help the natal group due to inclusive fitness (Jennions & Macdonald, 1994; Jimenez et al., 2017; Wright, 1951). These results are consistent with predictions for cooperative breeders, where reduced competition facilitates dispersal (Kokko & Lundberg, 2001; Maag et al., 2018). Conspecific density therefore appears to be a major factor influencing patterns of natal emigration.

In addition to density, prey availability also influenced emigration. Wolves originating from territories with high prey biomass were more likely to emigrate, potentially reflecting the advantages of high‐quality natal territories. Although prey biomass for a given GMU appeared relatively stable across years (Figure S6), substantial spatial heterogeneity and some variation for specific GMUs were observed in Idaho. These differences likely shape the natal conditions experienced by wolves and influence the probability of emigration. Contrary to our hypothesis, these results suggest that wolves originating from resource‐rich natal territories were more likely to emigrate. Wolves from resource‐rich natal territories might develop better physical condition, have enhanced reproductive potential and have a greater capacity to offset the fitness costs of dispersal (Bowler & Benton, 2009; Forero et al., 2002), allowing them to seek settlement opportunities elsewhere. This may be particularly true in males where larger body size could conceivably increase the likelihood of usurping a breeder and obtaining a breeding position in an established group (SunderRaj et al., 2022). Exposure to higher prey biomass can also provide an advantage by fostering learned skills (e.g. hunting prey) that facilitate successful dispersal. This result contrasts with studies linking dispersal to resource scarcity (Gese et al., 1996; Mech et al., 1998; Messier, 1985; Peterson et al., 1998) and suggests that heterogeneous landscapes with high‐quality natal territories may contribute disproportionately to disperser production (Bowler & Benton, 2009; Kokko & Lundberg, 2001; Lambin et al., 2001).

Contrary to our hypothesis and previous studies, harvest did not significantly influence natal dispersal in our system, indicating that its effects may vary across contexts or spatial scales. Adams et al. (2008) found that wolf populations in heavily harvested systems regulated their numbers by increasing dispersal patterns, such as emigration and immigration, when harvest rates were below 29%. This suggests that dispersal plays a key role in the recovery and regulation of wolf populations under human exploitation. However, the impact of harvest on wolf populations can vary. For example, Roffler et al. (2024) used long‐term genetic monitoring across an island system and found that harvest density in the natal area predicted the direction of wolf dispersal events. Wolves moved from areas of low harvest to areas with higher harvest density until a threshold was reached, above which they dispersed towards lower harvest areas. Our findings contradict this, suggesting that factors other than harvest, such as population density in the natal territory or kin selection, may play a more prominent role in shaping dispersal behaviour. This pattern is consistent with Bassing et al. (2020). Using data from Idaho between 2007 and 2016 under varying harvest intensities, Bassing et al. (2020) found that harvest did not significantly affect natal dispersal or settlement patterns and that immigration failed to offset harvest mortality. Overall, we found that harvest pressure did not affect the probability of emigration, indicating that intraspecific density and resource availability are stronger determinants of dispersal in our system.

Although sex was included in the top model, it was not included in an equally supported top model (Table 2). Our study did not find significant differences between sexes in emigration probability (*β* = 0.43, SE = 0.29, *p* = 0.14) or dispersal distance at harvest, although males represented 56% of dispersers. Interestingly, the longest observed dispersal event was by a female at 338 km, whereas the longest male dispersal was only 265 km (Figure S9). Rare long‐distance dispersal events by females have been documented in other wolf populations, including both colonizing populations in North America (Boyd & Pletscher, 1999) and the Scandinavian population, where a female wolf dispersed one of the longest known natal dispersal distances (>1000 km; Wabakken et al., 2007), and north‐western Europe (Andersen et al., 2015). More broadly, studies of a cooperative‐breeding bird show that the philopatric sex disperses less frequently but may undertake long‐distance movements when dispersal occurs (Kingma et al., 2017). However, the absence of a significant sex effect on emigration contrasts with many studies of North American wolves, which report male‐biased dispersal at the group level or sex differences in dispersal patterns, including distance (Boyd & Pletscher, 1999; Jimenez et al., 2017; Morales‐González et al., 2021). Male‐biased dispersal is typically observed because males leave natal territories to seek breeding opportunities, whereas females tend to remain philopatric (Ausband, 2022b). In our study, however, male and female wolves in Idaho under current conditions appear equally likely to emigrate beyond their natal territories, although our conservative dispersal distance threshold may have reduced detection of short‐distance emigration events. These findings suggest that sex‐biased patterns observed in other populations may emerge later during settlement or in group formation rather than in the emigration phase.

While our analysis provides key insights, several limitations also influence our interpretation of the results that highlight areas for further exploration in dispersal ecology. First, our study could not evaluate age‐related dispersal patterns because we did not know the exact age at which dispersal began. As a result, we relied on the studies showing that wolf pups rarely disperse (Jimenez et al., 2017; Morales‐González et al., 2021), although age‐related dispersal remains an important factor to consider for a more comprehensive understanding of dispersal dynamics. Second, when siblings <1 year‐of‐age are harvested in distant locations, the centroid we calculated may not fall at the true centre of their natal territory. However, by identifying dispersers beyond twice the radius of the average territory size, we likely avoided misclassifying individuals that remained near their natal territory, thereby ensuring a more conservative assessment of natal emigration. Still, wolf territories can vary substantially in size and shape, introducing additional uncertainty. Furthermore, our covariates for prey biomass, wolf density and harvest represent coarse metrics that may not fully capture the group‐level conditions influencing dispersal decisions. Finally, our study also lacked data on the transient stage of dispersal. Some individuals may have still been in transit when harvested, which could affect estimates of dispersal distance if final settlement had not yet occurred. However, this limitation is less likely to influence the classification of emigration status because individuals identified as emigrants had already moved beyond their natal territories. Future research should examine the costs and risks associated with dispersal during transience to improve understanding of settlement decisions and increase the accuracy of predictive models. Additionally, future studies that include larger sample sizes from sibling groups could include ‘natal group’ as a random effect to account for within group variability.

Understanding how population density and prey availability influence wolf emigration in Idaho can help inform management decisions. Areas with relatively high density and resources may produce disproportionate numbers of dispersers, supplying individuals to heavily harvested regions or newly colonized areas. Such movements may influence regional population structure and redistribution of individuals across the landscape. Monitoring prey availability in these areas may therefore improve managers' ability to anticipate emigration patterns.

To our knowledge, this study presents the first ecological application of genetic sibship to quantify dispersal patterns in a group‐living species. By integrating genetic and ecological data from harvest samples, we demonstrate the value of a novel genetic approach for examining emigration within a cooperative carnivore system. This framework provides a broadly applicable genetic method that uses kin structure to understand dispersal, and it offers insights not only for cooperative breeders but also for social species with structured family groups, including primates, birds and mammals. Insights from cooperative‐breeding theory can help guide the management and conservation of group‐living species, for which integrative approaches remain limited (Hing et al., 2017; Mech, 2020; Morales‐González et al., 2021).

## AUTHOR CONTRIBUTIONS

David E. Ausband, Matthew A. Mumma and Kathleen M. Petersen conceived the ideas and designed the methodology. Matthew A. Mumma, Joel M. Tebbenkamp and David E. Ausband collected the data. Joel M. Tebbenkamp conducted the sibship analyses. Kathleen M. Petersen analysed the data and led the writing of the manuscript. All authors contributed critically to the drafts and gave final approval for publication.

## CONFLICT OF INTEREST STATEMENT

The authors declare no conflicts of interest.

## STATEMENT ON INCLUSION

We shared the outcomes of this research through presentations at regional wildlife society conferences and meetings with state wildlife managers. We also contributed to a documentary film produced through a separate project to broaden accessibility and public engagement with stakeholders in the region where the study occurred.

## Supporting information


**Data S1.** Assigning harvest locations.
**Methods S2.** Dispersal distance calculations.
**Methods S3.** Relative abundance model parameters.
**Methods S4.** Prey biomass calculations.
**Methods S5.** Wolf harvest calculations.
**Figure S6.** Maps of estimated relative wolf abundance derived from camera trap data, in Idaho, USA, from 2017 to 2022. Each map (a–f) represents a survey area, with colour intensity indicating estimated wolf abundance within each 686 km^2^ cell.
**Figure S7.** Prey biomass (in kg/effort day/100 km^2^) estimated for grey wolves in Idaho, USA, from 2017 to 2022, scaled by 1 standard deviation (SD) of prey biomass. km^2^.
**Figure S8.** Grey wolf harvest intensity (wolves harvested per GMU per 1000 km^2^) in Idaho, USA, from 2017 to 2022.
**Figure S9.** Boxplot of apparent dispersal distances (in kilometres) of female and male grey wolves (*Canis lupus*) in Idaho, USA, from 2017 to 2023.
**Figure S10.** Plot showing odds ratios and 95% confidence intervals for probability of natal emigration of grey wolves (*Canis lupus*) in Idaho, USA, 2017–2023. Prey biomass, wolf density and harvest were standardized.
**Table S11.** Distances (in kilometres) and natal emigration status for grey wolves (*Canis lupus*) in Idaho, USA using assessed using harvest data from 2017 to 2023. Natal emigrants and non‐dispersers include all potential dispersers aged 1 year and older, while sexes reflect only those categorized as natal emigrants.
**Table S12.** Covariates from the linear model predicting grey wolf (*Canis lupus*) apparent dispersal distance in relation to sex in Idaho, USA, from 2017 to 2023. The table provides estimated coefficients (*β*), standard errors (SE) and *p*‐values (*p*) for each covariate.

## Data Availability

Data are available from the Zenodo Digital Repository: https://doi.org/10.5281/zenodo.21461372 (Petersen et al., 2026).
