# Supplementary material for: Understanding natal emigration in group‐living carnivores
Source: J Anim Ecol. 2026 Jul 30;95(10):2076–87. doi: 10.1111/1365-2656.70330 (PMC13643189; doi:10.1111/1365-2656.70330)
Supplement: Supplementary file 1 [file JANE-95-2076-s001.docx]

**Supplementary Information**

**Supplementary Methods**

Methods S1: Assigning Harvest Locations
We assigned geographic locations to harvested wolves by referencing kill locations reported by hunters and incorporating biologists' inferences for the predicted 686 km^2^ cell associated with a geographic feature. When locations spanned two geographic features, such as rivers or creeks, we calculated the midpoint to assign a representative harvest location.

Methods S2: Dispersal Distance Calculations

Territory size: The mean Idaho wolf territory size is 686 km² (Ausband et al. 2016), corresponding to a circular radius of 14.8 km. Dispersers were defined as wolves harvested more than 29.6 km (two times the radius) from their sibling or centroid location.

Methods S3: Relative Abundance Model Parameters

- Dates: June 15–September 20, with 7 sampling intervals of 14 days each.
- Detection criteria: Independent detections were defined as ≥20 minutes apart and used the max count of wolves per detection event.

For both start and end locations, it was noted that some estimates were missing for specific cells in certain years. In cases where abundance estimates were missing, the corresponding cell was replaced with the closest available abundance estimate from either the start or end latitude/longitude. This approach ensured that the analysis reflects the abundance estimate of the nearest neighbor cell.

Methods S4: Prey Biomass Calculations

- Not all GMUs have all three species (white-tailed deer, mule deer, elk) available for general hunting.
- GMU Area is constant through years.
- Prey biomass values:
  - Elk: 192.44 kg – female and male mean (Greer & Howe 1964)
  - Mule deer: 61.80 kg – female and male mean (Mackie 1964)
  - White-tailed deer: 51.19 kg—study mean (Silver et al. 1959).
- Equations:
  - CPUE _year,species,GMU_ = Harvested Animals / Days hunted
  - Biomass CPUE=CPUE × Biomass (kg)
  - Adjusted CPUE= Biomass CPUE / GMU Area (km^2^) × 100

Methods S5: Wolf Harvest Calculations

- Equations:
  - Harvest_year,GMU_ = Harvested wolves per Game Management Unit per Year
  - Harvest_year(Adjusted)_ = Harvested wolves / GMU Area (km^2^) × 1,000

**Supplementary Figures**

S6. Maps of estimated relative wolf abundance derived from camera trap data, in Idaho, USA , from 2017 to 2022. Each map (a-f) represents a survey area, with color intensity indicating estimated wolf abundance within each 686 km^2^ cell.

Figure S7. Prey biomass (in kg/effort day/100 km²) estimated for gray wolves in Idaho, USA, from 2017 to 2022, scaled by 1 standard deviation (SD) of prey biomass. km^2^.

Figure S8. Gray wolf harvest intensity (wolves harvested per GMU per 1,000 km²) in Idaho, USA, from 2017 to 2022.

Figure S9. Boxplot of apparent dispersal distances (in kilometers) of female and male gray wolves (*Canis lupus*) in Idaho, USA, from 2017 to 2023.

Figure S10. Plot showing odds ratios and 95% confidence intervals for probability of natal emigration of gray wolves (*Canis lupus*) in Idaho, USA, 2017–2023. Prey biomass, wolf density and harvest were standardized.

**Supplementary Tables**

Table S11. Distances (in kilometers) and natal emigration status for gray wolves (*Canis lupus*) in Idaho, USA using assessed using harvest data from 2017–2023. Natal emigrants and non-dispersers include all potential dispersers aged one year and older, while sexes reflect only those categorized as nata emigrants.

| Class | Mean distance |  | Min. distance | Max distance | n |
| --- | --- | --- | --- | --- | --- |
| Non-Disperser | — |  | — | — | 138 |
| Natal emigrant | 81.8 |  | 30 | 338.9 | 86 |
| Female | 81.8 |  | 30 | 338.9 | 38 |
| Male | 81.9 |  | 31.7 | 264.7 | 48 |

Table S12. Covariates from the linear model predicting gray wolf (*Canis lupus*) apparent dispersal distance in relation to sex in Idaho, USA, from 2017 – 2023. The table provides estimated coefficients (*β*), standard errors (SE), and p-values (*p*) for each covariate.

| Covariate | *β* | SE | *p* |
| --- | --- | --- | --- |
| Intercept | 0.35 | 0.05 | <0.001 |
| Male | 0.08 | 0.07 | 0.25 |
|  |  |  |  |
